# CD36-mediated podocyte lipotoxicity promotes foot process effacement

**DOI:** 10.1515/med-2024-0918

**Published:** 2024-03-19

**Authors:** Wei Hua, Lan Peng, Xue-mei Chen, XuShun Jiang, JianGuo Hu, Xian-Hong Jiang, Xu Xiang, Jiangmin Wan, Yingfei Long, Jianqiong Xiong, Xueyi Ma, Xiaogang Du

**Affiliations:** Department of Nephrology, The First Affiliated Hospital of Chongqing Medical and Pharmaceutical College, Chongqing 400000, China; Basic Department, Chongqing Medical and Pharmaceutical College, Chongqing 401331, China; Emergency Department, The First Affiliated Hospital of Chongqing Medical University, Chongqing 400042, China; Department of Nephrology, The First Affiliated Hospital of Chongqing Medical University, Chongqing 400042, China; Department of Obstetrics and Gynecology, Second Affiliated Hospital, Chongqing Medical University, Chongqing, China; Department of Nephrology, People’s Hospital of Qijiang District, Chongqing 401420, China; Department of Obstetrics and Gynecology, The Third Affiliated Hospital of Chongqing Medical University, Chongqing, 401120, China; Hospital of Chongqing University, Chongqing, 400044, China; Department of Nephrology, The First Affiliated Hospital of Chongqing Medical University, Youyi Road 1, Chongqing 400042, China

**Keywords:** CD36, podocyte, foot process effacement, palmitic acid, ROS

## Abstract

**Background:**

Lipid metabolism disorders lead to lipotoxicity. The hyperlipidemia-induced early stage of renal injury mainly manifests as podocyte damage. CD36 mediates fatty acid uptake and the subsequent accumulation of toxic lipid metabolites, resulting in podocyte lipotoxicity.

**Methods:**

Male Sprague-Dawley rats were divided into two groups: the normal control group and the high-fat diet group (HFD). Podocytes were cultured and treated with palmitic acid (PA) and sulfo-*N*-succinimidyl oleate (SSO). Protein expression was measured by immunofluorescence and western blot analysis. Boron-dipyrromethene staining and Oil Red O staining was used to analyze fatty acid accumulation.

**Results:**

Podocyte foot process (FP) effacement and marked proteinuria occurred in the HFD group. CD36 protein expression was upregulated in the HFD group and in PA-treated podocytes. PA-treated podocytes showed increased fatty acid accumulation, reactive oxygen species (ROS) production, and actin cytoskeleton rearrangement. However, pretreatment with the CD36 inhibitor SSO decreased lipid accumulation and ROS production and alleviated actin cytoskeleton rearrangement in podocytes. The antioxidant N-acetylcysteine suppressed PA-induced podocyte FP effacement and ROS generation.

**Conclusions:**

CD36 participated in fatty acid-induced FP effacement in podocytes via oxidative stress, and CD36 inhibitors may be helpful for early treatment of kidney injury.

## 1 Introduction

Lipid metabolism disorders, which contribute to atherosclerosis, are also emerging as risk factors for the progression of renal disease [1,2]. Hyperlipidemia is believed to contribute to the onset of renal injury through the glomerular accumulation of lipids [3]. A number of models of experimental renal disease, as well as renal biopsies from patients with renal diseases, which focused mostly on mesangial cells and macrophages, found lipid and apolipoprotein deposition. The conversion of mesangial cells to foam cells plays an important role in the development of glomerulosclerosis through the acquisition of lipids [1]. However, a previous study confirmed that the early stage of renal injury induced by lipid metabolism disorder mainly involved podocyte damage [2]. Lipid accumulation in the kidney leads to dysfunction and lipotoxicity in podocytes, including endoplasmic reticulum stress, oxidative stress, and foot process (FP) effacement [3].

Podocytes are a type of intrinsic glomerular cell and specialized epithelial cell located on the visceral side of the glomerulus. Podocytes are an integral part of the renal glomerular filtration barrier [4]. These cells consist of three segments: the cell body, major processes, and FPs. The unique shape of the podocyte FP is derived from a rich actin cytoskeleton, which serves as the podocyte backbone. The FPs can adhere to the glomerular basement membrane (GBM) with the help of the actin cytoskeleton. Moreover, FPs interdigitate through intercellular junctions, called slit diaphragms (SDs), to prevent proteinuria [5]. Moreover, the actin cytoskeleton enables podocytes to continually and dynamically alter their shape and serves as a static function. Therefore, damage to the actin cytoskeleton could lead to FP effacement and eventually lead to proteinuria as renal glomerular filtration membrane integrity is destroyed. Saturated fatty acids (palmitic acid, PA) can affect cytoskeletal rearrangement and induce severe morphological changes in neurons [6]. In addition, podocyte lipotoxicity affects the rearrangement of the actin cytoskeleton and the simplification of FP interdigitation, which is called FP effacement and is the common pathway of all proteinuria-associated diseases [3]. However, the mechanisms underlying the effects of fatty acids on cytoskeletal rearrangement and FP effacement are not clear.

CD36, a class B transmembrane scavenger receptor, plays a crucial role in the regulation of lipid metabolism and signaling. CD36 also causes lipotoxicity in multiple cell types. MiR-320 induces lipotoxicity in cardiomyocytes by activating CD36 transcription, which is responsible for increased fatty acid uptake, thereby causing cardiac dysfunction [7]. PA mediates endoplasmic reticulum stress and lung epithelial cell death via the upregulation of CD36, leading to lung injury and fibrosis [8]. Excessive production of oxidative proteins promotes lipotoxicity via the CD36/β-catenin pathway, mediating lipid accumulation and inducing injuries to proximal tubular epithelial cells and tubulointerstitial fibrosis [9]. CD36 has been proposed to be a major endocytic receptor for fatty acids in podocytes [10], and CD36-mediated excess lipid accumulation induces oxidative stress and podocyte apoptosis [11].

In this study, it was assumed that PA induced CD36 expression, lipid absorption, cytoskeletal rearrangement, and FP effacement via oxidative stress. Therefore, SSO was used to inhibit the fatty acid accumulation mediated by CD36. It showed that SSO rescue PA-induced cytoskeletal rearrangement and FP effacement. To gain further insight into the mechanisms by which CD36 mediates PA-induced alterations in podocyte morphology, reactive oxygen species (ROS) generation was suppressed with the potent antioxidant N-acetylcysteine (NAC) to alleviate PA-induced podocyte FP effacement. This study showed that CD36-mediated oxidative stress was implicated in PA-induced FP effacement and may act as a novel therapeutic target for treating kidney disease accompanied by hyperlipidemia.

## 2 Materials and methods

### 2.1 Experimental animals and measurement of general parameters

A total of 20 male Sprague-Dawley (SD) rats, aged 6–8 weeks, were obtained from the Chongqing Medicine University Medical Laboratory Animal Center and housed in a room at 22–25°C with 50–60% relative humidity and a 12 h light/dark cycle. The rats were randomized into the following two groups: the normal control group (NC), which was provided basic feed, and the high-fat diet group (HFD), which was provided a high-fat diet (2% cholesterol, 0.3% deoxysodium cholate, 7.5% lard, 0.2% propylthiouracil, 5% egg yolk powder, and 85% basal feed). The body weights of rats were recorded every week. Blood, urine, and renal tissues were harvested after 4 and 10 weeks. The levels of blood lipids (total cholesterol [TC], triglyceride [TG], and low-density lipoprotein cholesterol [LDL-c]) were tested using a fully automatic biochemical analyzer. The rats were kept alone in metabolic cages, and 24 h urine samples were collected to quantitatively analyze the level of 24 h urinary protein using Coomassie brilliant blue staining. Glomerular morphology was observed using an electron microscope, and protein was extracted to measure CD36 expression using western blot analysis.

### 2.2 Podocyte culture and treatments

Conditionally immortalized mouse podocyte cell line was shared by University College Medical School, London, United Kingdom and Dr Ruan of the Centre for Nephrology, Royal Free. Podocytes were cultured under permissive conditions (33°C) in Roswell Park Memorial Institute 1640 supplemented with 10% fetal bovine serum (Gibco) and 10 U/mL interferon-γ on type I collagen. Differentiation was induced by changing the temperature to 37°C in the absence of interferon-γ [12]. All experiments were performed on podocytes that had been differentiated for 14 days. Palmitate (5 mmol/L) was diluted with 10% bovine serum albumin/phosphate-buffered saline (BSA/PBS) (w/v) for 24 h at room temperature, producing a 5 mmol/L free fatty acid (FFA)/10% BSA mixture. The 5 mmol/L FFA/10% BSA mixture was diluted to different ratios with serum-free culture medium to the intended final concentrations (50–300 µmol/L) of palmitate. The vehicle control for palmitate was 1% BSA. Podocytes were treated with or without SSO (50 µmol/L; Toronto Research Chemicals), NAC, or vehicle (dimethyl sulfoxide, DMSO) for 4 h prior to treatment with palmitate (150 µmol/L; Sigma–Aldrich) or vehicle (5% BSA) for an additional 4 h. CD36 expression was measured by western blot and immunofluorescence analysis, lipid uptake was measured by boron-dipyrromethene (BODIPY) staining, ROS production was measured by 2′,7′-dichlorofluorescein diacetate (DCFH-DA) staining, and the actin cytoskeleton in podocytes was examined by rhodamine phalloidin staining.

### 2.3 Electron microscopy

Electron microscopy was performed to examine the ultrastructure of kidney tissue. After fixation with 4% glutaraldehyde for several hours at 4℃, kidney tissue was postfixed with 1% osmium tetroxide. The samples were dehydrated in a graded series of acetone. Then, the tissue was embedded in Durcupan and sliced with an ultramicrotome. Ultrathin sections were observed with a JEM-1200 EXII transmission electron microscope (JEOL, Tokyo, Japan).

### 2.4 Measurement of podocyte FP width (FPW)

Glomeruli from each specimen were observed by transmission electron microscopy (original magnification ×10,000). In each photograph, the GBM was measured by Image-Pro Plus 6.0 software. The GBM and the number of podocyte FPs were counted and computed. Then, the average FPW was calculated with the computational formula [13]:

\[\text{FPW}=\pi/\text{4}\sum \text{GBM}\hspace{.25em}\text{length}/\sum \text{FP}\text{,}]\]

where ∑GBM length represents the total GBM length in each image, and ∑FP represents the total number of FPs in each image.

### 2.5 Immunocytochemistry

Tissue staining was accomplished on paraffin vertical sections using antibodies against CD36 and nephrin. Briefly, after deparaffinization, rehydration, antigen recovery, and blocking, the sections were incubated with rabbit polyclonal anti-CD36 antibody (1:200; Novus Biologicals) and mouse monoclonal anti-nephrin antibody (1:200; Santa) overnight at 4°C. Sections were washed extensively with PBS and incubated with secondary antibodies (goat anti-rabbit-IgG and goat anti-mouse-IgG, 1:200; Servicebio) and 4′,6-diamidino-2-phenylindole (2 μg/mL; Invitrogen) for 2 h at room temperature [14].

### 2.6 Protein extraction and **w**estern blot analysis

Cells were rinsed twice with PBS, sonicated for 15 s in 500 μL of RIPA lysis buffer (Beyotime, China), and centrifuged at 14,000*g* for 5 min. Protein concentration was then determined by bicinchoninic acid protein assay (Beyotime, Beijing, China). The sample loading buffer was added to the protein sample and heated at 100°C for 10 min. The proteins (20 μg/well) were separated by electrophoresis on 10% sodium dodecyl sulfate–polyacrylamide gel electrophoresis gels. After transfer, the membranes were incubated with rabbit monoclonal CD36 antibodies (1:800, ab133625; Abcam). Horseradish peroxide-conjugated goat anti-rabbit immunoglobulin and chemiluminescence were used for detection [15]. β-actin served as a loading control.

### 2.7 Measurement of lipid uptake

Lipid uptake was measured by oil red staining in kidney tissue and BODIPY staining in podocytes. The lipid deposition in kidney tissue was determined by Oil Red O [16]. Podocytes were incubated with BODIPY dye (10 µg/mL, BODIPY 500/510 C1, C12; Invitrogen, USA) at 37°C for 2 h, and then the cells were washed with PBS to remove extracellular fatty acids. The images were visualized by an ordinary light microscope and a fluorescence microscope.

### 2.8 F-actin labeling

Rhodamine phalloidin (3.5:500, Cat. PHDR1; Cytoskeleton Inc., USA) was used to examine the actin cytoskeleton in podocytes. Podocytes were fixed with 4% paraformaldehyde at room temperature for 10 min and then washed with PBS for 30 s. Then, the podocytes were treated with permeabilization buffer (0.2% Triton X-100 in PBS) at room temperature for 10 min. After being rinsed, the podocytes were incubated with rhodamine phalloidin (diluted in PBS according to the manufacturer’s specifications) in the dark at room temperature for 20 min. The cells were then rinsed in PBS and observed under a fluorescence microscope [17].

### 2.9 Statistical analysis

Statistical analysis was performed by SPSS 22.0. All determinations were based on three replicate samples. Quantitative data are presented as the mean ± standard deviation. Statistical significance between groups was analyzed by Student’s *t*-test and one-way analysis of variance. Statistical significance was set at *P* < 0.05.


**Ethics approval and consent to participate**: The study was carried out after the protocol was approved by the ethics committee The First Affiliated Hospital of Chongqing Medical University, approval number 2015-63. The authors confirm that all methods were performed in accordance with the relevant guidelines. All procedures were performed in accordance with the ethical standards laid down in the 1964 Declaration of Helsinki and its later amendments.

## 3 Results

The theme of this study was to explore the relationship between dyslipidemia and podocyte FP effacement, and the mechanism in this process. Moreover, this study aimed to show that PA induced CD36 expression, lipid absorption, cytoskeletal rearrangement, and FP effacement. We found that SSO, a specific inhibitor of fatty acid translocase (FAT)/CD36 disrupted lipid rafts, decreased fatty acid accumulation and rescued PA-induced cytoskeletal rearrangement and FP effacement, showing that CD36-mediated lipotoxicity participated in PA-induced actin cytoskeleton disruption. To demonstrate that oxidative stress was involved in lipotoxicity-induced FP effacement mediated by CD36, ROS generation was suppressed with the potent antioxidant NAC to alleviate PA-induced podocyte FP effacement. This study showed that CD36-mediated oxidative stress was implicated in PA-induced FP effacement.

### 3.1 Podocyte FP effacement and marked proteinuria occurred in the HFD group

**Figure 1:**
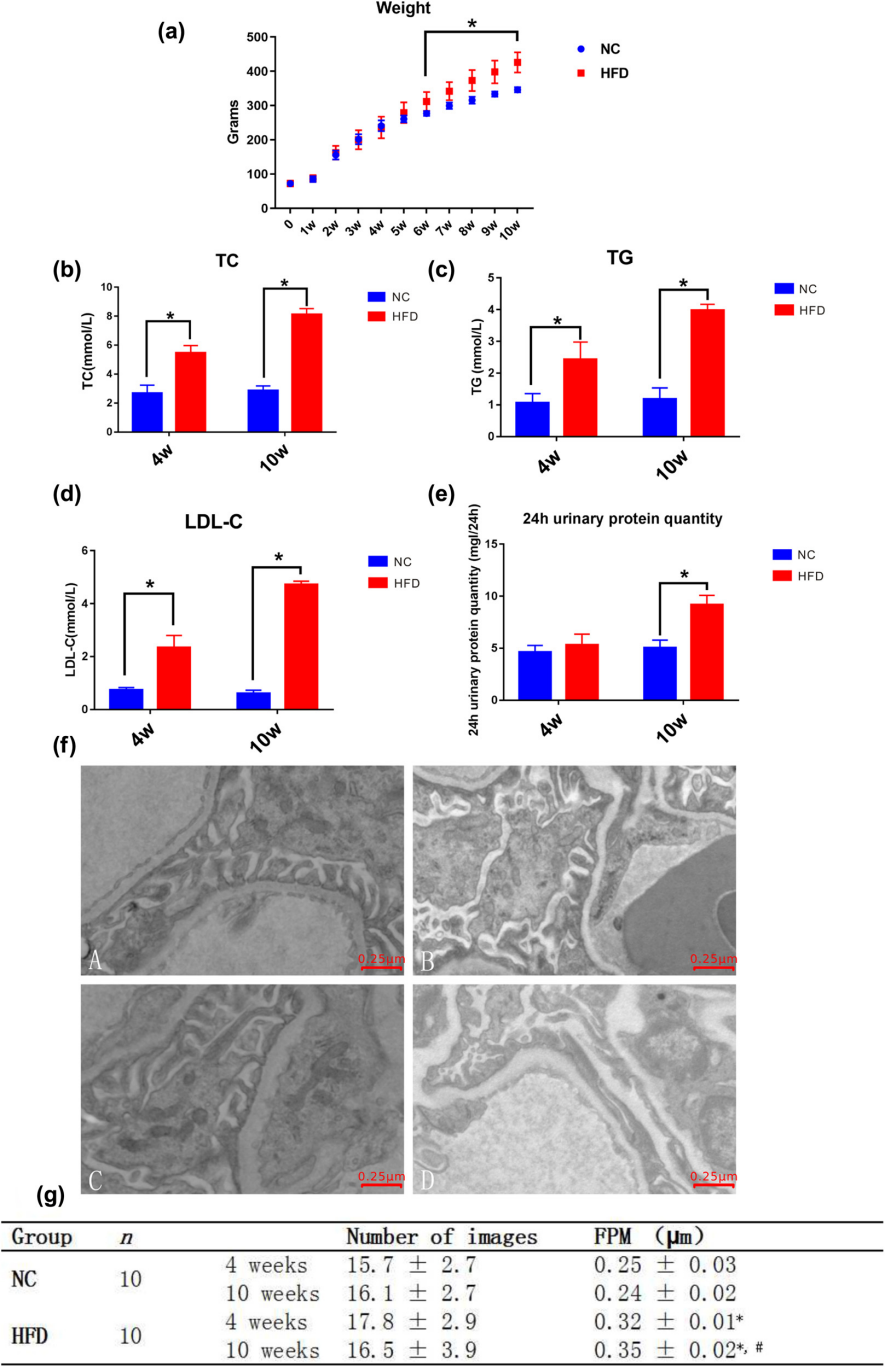
Podocyte FP effacement and a marked increase in proteinuria in response to a high-fat diet. (a)–(e) Comparison of the general indicators in the NC and HFD groups. Each group consisted of ten rats (*n* = 10). The data are presented as the mean ± standard deviation. (a)–(d) Comparison of the body weight of rats, the levels of TC, TG, and LDL-c in the two groups of rats. (e) Levels of 24 h urine protein of rats in metabolic cages were determined by the Coomassie brilliant blue staining method. *P < 0.05 vs NC group. (f) Transmission electron microscopy images showing the FP structure at different times (images were acquired at ×5,000). (a) The NC group was given basic feed for 4 weeks. (b) The HFD group was fed a high-fat diet (2% cholesterol, 0.3% deoxysodium cholate, 0.2% propylthiouracil, 7.5% lard, 5% egg yolk powder, and 85% basal feed) for 4 weeks. (c) The NC group was given basic feed for 10 weeks. (d) The HFD group was fed a high-fat diet (2% cholesterol, 0.3% deoxysodium cholate, 0.2% propylthiouracil, 7.5% lard, 5% egg yolk powder, and 85% basal feed) for 10 weeks. Representative images are shown. (g) Comparison of the FPW in the two groups. *P < 0.05 vs NC group; ^#^ *P* < 0.05 vs HFD group (4 weeks).

The body weights of rats were recorded every week. Urinary albumin excretion and blood lipid levels, including TG, TC, and LDL-c levels, were measured after 4 and 10 weeks of treatment with a high-fat diet (*n* = 10) or basic diet (*n* = 10). After 4 weeks, blood lipid levels and urinary albumin excretion were significantly higher in the HFD group than in the NC group. Furthermore, the HFD group showed further increases in blood lipid levels and urinary albumin excretion after 10 weeks compared with those after 4 weeks (*P* < 0.05) (Figure 1a–e).

Transmission electron microscopy demonstrated that in response to hyperlipidemia, the HFD group had a more significant degree of podocyte FP fusion than the NC group. As shown in Figure 1f, some podocytes in the glomerulus exhibited mild fused FPs in the HFD group after 4 weeks. After 10 weeks, FPs were extensively fused, bent, and thickened, and there was cellular edema in this group. Podocytes showed variable FP effacement in response to hyperlipidemia. Figure 1g shows the quantitative analysis of the extent of FP effacement as measured by the mean FPW. After 4 weeks, the mean FPW in the HFD group was wider than that in the NC group (0.32 ± 0.01 vs 0.25 ± 0.03 µm; *P* < 0.05). After 10 weeks, the FPW was significantly wider in the HFD group than that in the NC group (*P* < 0.05), and the degree was more serious than that at 4 weeks.

### 3.2 High expression of CD36 in the kidney tissue of the HFD group and podocytes treated with PA

**Figure 2:**
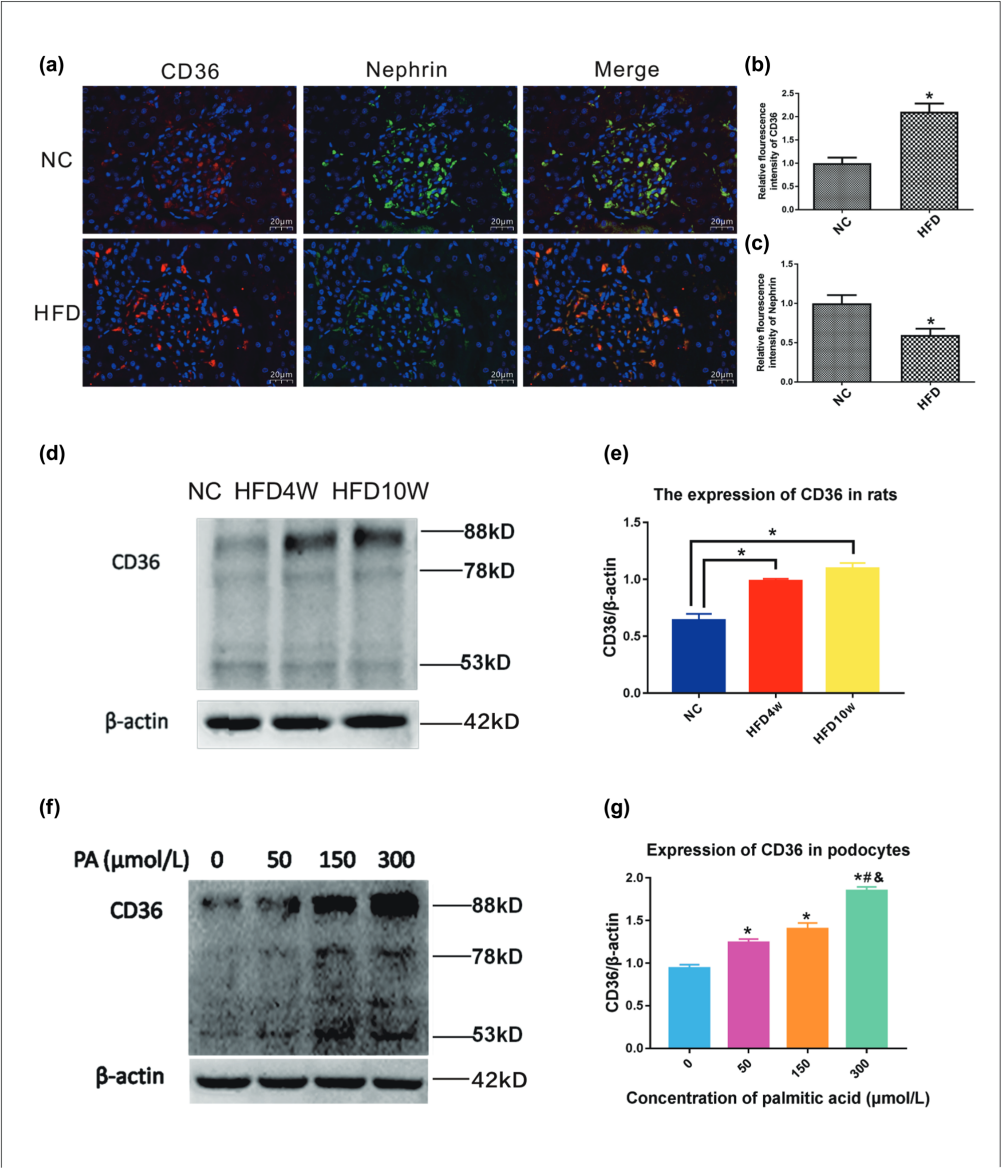
High expression of CD36 in the kidney of rats fed with high-fat diet and in podocytes treated with PA. (a) Immunofluorescence staining of CD36 and nephrin in renal tissue derived from control subjects or high-fat diet rats (×400). NC: the normal control group that was given basic feed for 10 weeks, HFD: the high-fat diet group that was provided a high-fat diet for 10 weeks. (b) and (c) Quantification of the fluorescence intensity of CD36 and nephrin staining (*n* = 5, *P < 0.05 vs NC group). (d) The western blot of CD36 expression in the kidney of rats. NC: the normal control group that was given basic feed for 4 weeks, HFD4W: the group that was fed a high-fat diet for 4 weeks, HFD10W: the group that was fed a high-fat diet for 10 weeks. (e) Densitometric analysis of CD36 (*n* = 3, *P < 0.05 vs NC group). (f) The western blot of CD36 expression in the podocytes. Podocytes were incubated with different concentrations of PA (0–300 µmol/L) for 12 h; CD36 expression was measured by western blot analysis (53, 78, and 88 kDa). β-actin was used as an internal control. Representative images are shown. (g) Densitometric analysis of CD36 (*n* = 3, *P < 0.05 vs 0 μmol/L, #*P* < 0.05 vs 50 μmol/L, ^&^ *P* < 0.05 vs  μmol/L).

CD36, which is a scavenger receptor, promotes long-chain fatty acid (LCFA) uptake [18]. Upon exposure to a HFD, CD36 levels were significantly increased in the livers of rats [19]. The CD36 expression was analyzed using immunofluorescence staining and western blotting. As shown in Figure 2a, the expression of CD36 in both renal tubules and glomerulus of HFD group increased significantly when compared with the NC group. In contrast, the nephrin, a podocyte-specific transmembrane protein, was decreased in the HFD group compared with the NC group, which is a hallmark of podocyte injury. In glomerulus, CD36 was co-located with nephrin, suggesting that CD36 was expressed in podocytes. Kidney tissue samples were collected from ten NC rats and ten HFD rats, and CD36 protein expression was analyzed by western blotting to further verify the increased expression of CD36 in the kidneys in the HFD group. As shown in Figure 2b and c, CD36 levels were increased in the HFD group compared with the NC group. After 10 weeks, CD36 expression in the kidney was markedly higher in the HFD group than in the NC group. When fat accumulates, lipids are primarily stored in the cytoplasm as TG. Moreover, PA, which is a saturated LCFA, is one of the most important constituents of TG and plays a crucial role in the development of lipotoxicity [20]. A previous study examined whether PA could upregulate CD36 expression in podocytes [11]. Podocytes were treated for 12 h with different concentrations of PA (0–300 µmol/L) to further demonstrate that the CD36 protein increased in a dose-dependent manner. As shown in Figure 2d and e, western blot analysis was performed on podocytes and showed the high expression of CD36 in response to PA induction.

### 3.3 CD36-mediated lipid uptake and cytoskeletal rearrangement in podocytes under PA induction

**Figure 3:**
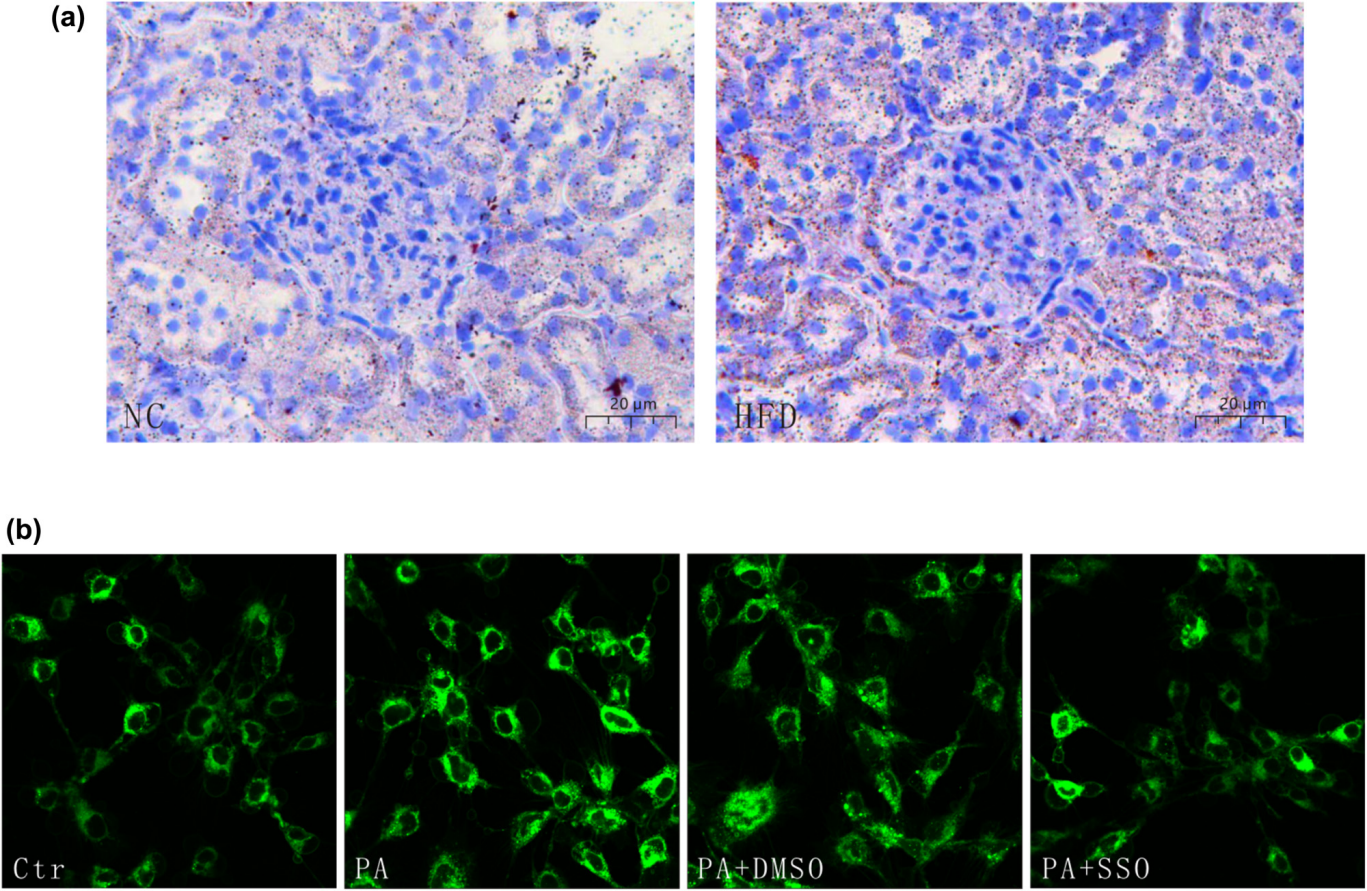
CD36 mediated lipid uptake under PA induction. (a) Lipid accumulation in control subjects or high-fat diet rats are stained with Oil Red O (×400). (b) Representative immunofluorescence images of podocytes stained with BODIPY (×300). Ctr: control group, in which podocytes were treated with 1% BSA. PA: PA group, in which podocytes were treated with 150 µmol/L PA for 12 h. PA + DMSO: podocytes were treated with 150 µmol/L PA for 12 h after being pretreated with DMSO for 4 h. PA + SSO: podocytes were treated with 150 µmol/L PA for 12 h after being pretreated with 50 µmol/L SSO for 4 h.

CD36 is an integral transmembrane glycoprotein that is expressed in various tissues, including the kidney, where it is involved in LCFA uptake. Upregulation of CD36 induces excess lipid accumulation in the kidney, stimulating cellular lipotoxicity [16]. SSO, a known inhibitor of FAT/CD36, prevents LCFA transport into cells [21,22]. In this study, we found that high-fat diet would induce lipid accumulation in the kidney (Figure 3a). To prove the key role of CD36 in the process of lipid uptake, podocytes were pretreated with SSO, the specific inhibitor of CD36, and then stimulated with PA, and the increased lipid uptake by podocytes under PA induction was disrupted. This result indicated that high-lipid conditions promoted CD36-mediated lipid uptake into podocytes (Figure 3b).

### 3.4 Oxidative stress participated in podocyte FP effacement mediated by CD36 upregulation in response to PA

**Figure 4:**
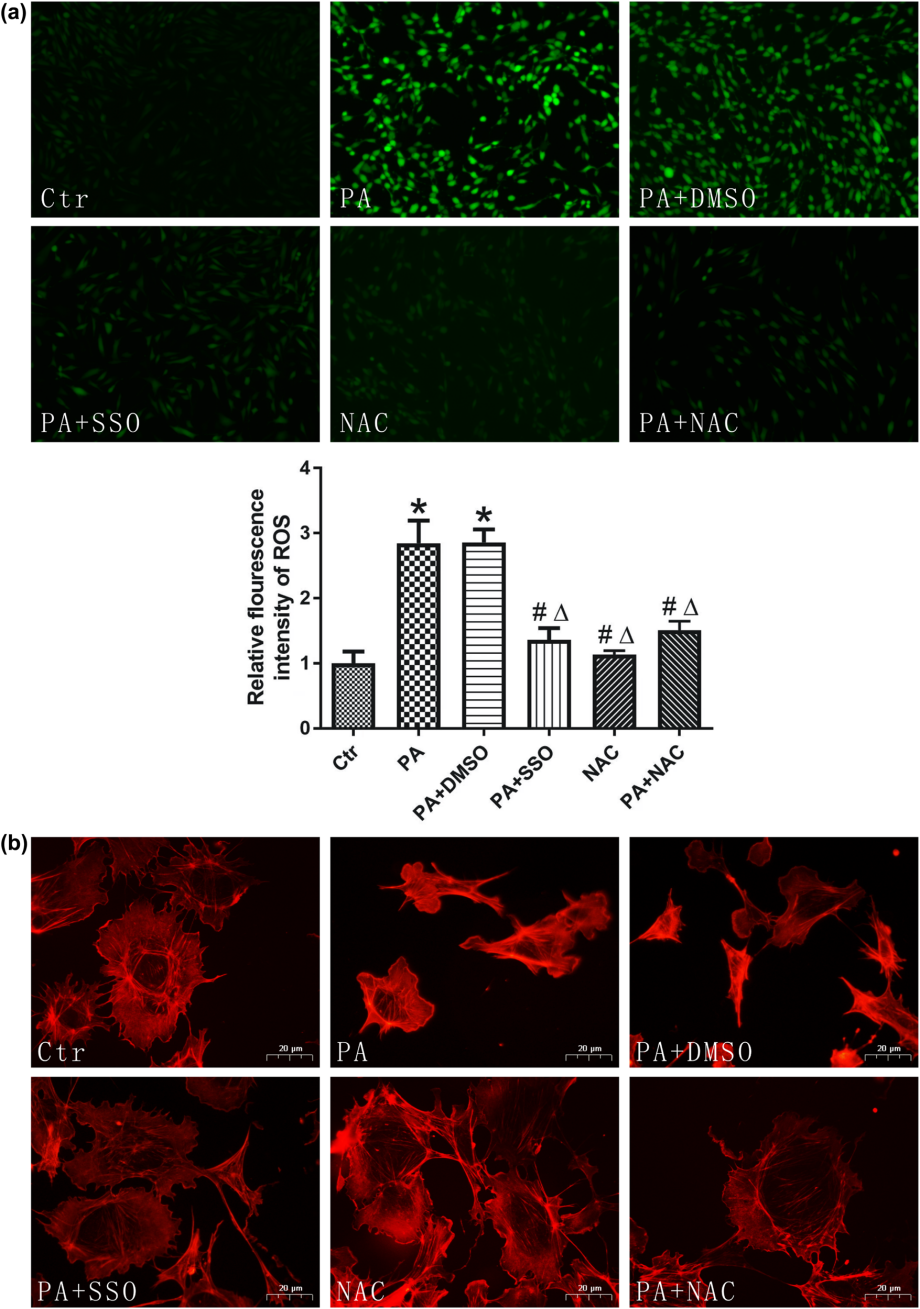
Oxidative stress participated in CD36-mediated podocyte FP effacement induced by PA. (a) The images and fluorescence intensity of intracellular ROS stained by the fluorescent probe DCFH-DA. *P < 0.05 vs Ctr group, ^#^ *P* < 0.05 vs PA group, Δ*P* < 0.05 vs PA + DMSO group. (b) Podocytes were treated with 150 µmol/L PA for 12 h with or without NAC or SSO pretreatment, and podocytes were stained with Alexa Fluor 568-labeled phalloidin. Ctr: control group, in which podocytes were treated with 1% BSA. PA: PA group, in which podocytes were treated with 150 µmol/L PA for 12 h. PA + DMSO: podocytes were treated with 150 µmol/L PA for 12 h after being pretreated with DMSO for 4 h. PA + SSO: podocytes were treated with 150 µmol/L PA for 12 h after being pretreated with 50 µmol/L SSO for 4 h.

Oxidative stress is one of the key factors associated with cell injury induced by high levels of fatty acids. ROS were measured by the ROS-sensitive fluorescent probe DCFH-DA to monitor cellular oxidative stress. The results showed that compared with that in the control group, ROS production was significantly increased in PA-induced podocytes. Then, the function of CD36 on podocytes was inhibited with SSO, followed by treatment with PA. ROS production decreased compared with that in the PA group and the PA + DMSO group (Figure 4a), demonstrating that oxidative stress induced by fatty acids in podocytes was mediated by CD36. Intracellular lipid accumulation in podocytes promotes the rearrangement of the actin cytoskeleton [3]. The actin cytoskeleton maintains normal podocyte shape [21], and FP effacement is the most prominent change in shape [22]. Furthermore, ROS could induce actin filament polymerization, leading to cytoskeletal dysfunction and structural changes and altering podocyte FPs [23]. Therefore, oxidative stress might be involved in FP effacement caused by podocyte lipotoxicity via CD36.

**Figure 5:**
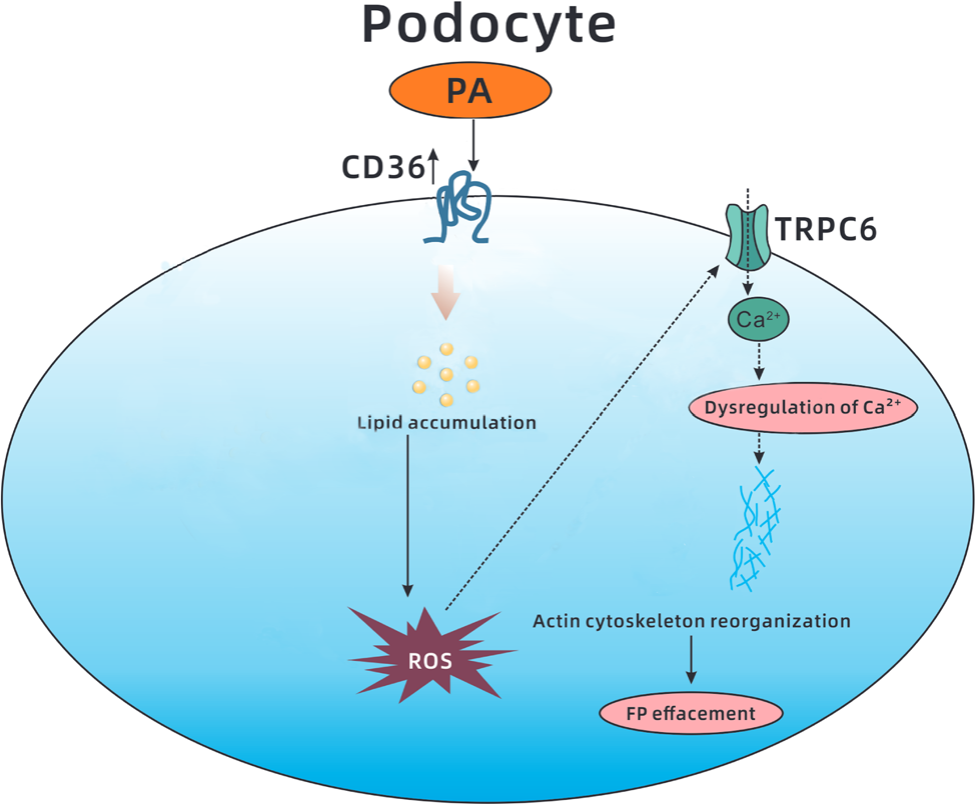
Hypothetical mechanism for PA-induced podocyte FP effacement. PA exposure upregulates CD36 expression and then induces an abnormal accumulation of lipids, further promoting oxidative stress. Excessive ROS generation activates actin reorganization and ultimately results in podocyte FP effacement, which may occur through TRPC6 channel-activated dysregulation of intracellular Ca^2+^ in podocytes.

Rhodamine phalloidin was used to label the actin cytoskeleton (red color) to assess the lipotoxicity and changes in cytoskeletal structure in PA-induced podocytes. Podocytes were pretreated with SSO, followed by treatment with PA. F-actin staining showed that PA disrupted the uniformly organized actin stress fibers, resulting in a morphological change in podocytes that was characterized by FP spreading, which is known as effacement [24]. However, SSO rescued PA-induced actin cytoskeleton rearrangement and FP effacement by inhibiting CD36-mediated lipid uptake in podocytes. Moreover, the potent antioxidant NAC suppressed podocyte FP effacement and ROS generation induced by PA (Figure 4b). These results showed that oxidative stress participated in CD36-mediated podocyte FP effacement induced by PA (Figure 5).

## 4 Discussion

Podocytes are intrinsic glomerular cells that are divided into three morphologically distinct segments: the cell body, major processes, and FPs. The FPs, which are the last barrier of the glomerular filtration barrier, interdigitate with neighboring FPs through SDs to prevent the development of proteinuria and blood plasma protein loss [5]. FPs consist of three domains: the apical membrane domain, the basal membrane domain and the SD, which are physically linked to the FP actin cytoskeleton [25]. The unique shape of the podocyte FP derives from an abundantly rich actin cytoskeleton, which serves as the podocyte’s backbone. Therefore, the morphology of the FP is based largely on the maintenance of highly ordered, parallel, and contractile actin filament bundles [23]. The rearrangement or dysfunction of the actin cytoskeleton leads to the retraction and loss of actin-based FPs, which is known as FP effacement [26] and ultimately results in proteinuria.

Hyperlipidemia is associated with the prevalence of metabolic syndrome, including the development of renal disease. A large amount of data indicates that in renal disease, hyperlipidemia plays a crucial role in accelerating glomerular and interstitial damage. Clinical studies also showed that renal function declined more rapidly among patients with primary renal disease or diabetic nephropathy who had hyperlipidemia [27]. Recent studies have shown that podocytes are a key factor in the pathogenesis and progression of hyperlipidemia-induced renal injury [11,28,29]. In this study, a mild degree of podocyte FP effacement was observed in rats fed a high-fat diet for 4 weeks. However, after 10 weeks, serious podocyte injury indicated by FP effacement and collapse was observed in the HFD group; moreover, a reduction in podocyte FP density, which was due to the increased level of blood lipids, was observed. The FPW, as an index to quantitatively assess FP effacement, was increased and more extensive in the HFD group than in the NC group. This result was consistent with that of a previous study [30,31]. Furthermore, total proteinuria was measured in each group to evaluate the effect of high blood lipids on renal function. The rats that were fed a HFD for 10 weeks showed marked increases in urinary excretion, which was associated with severe podocyte FP effacement. However, the mechanisms of hyperlipidemia-induced FP effacement are not clear.

This study provided evidence that hyperlipidemia may promote FP effacement via CD36-mediated lipotoxicity. CD36 is a transmembrane protein in the class B scavenger receptor family that facilitates the transport of LCFAs into cells [19]. Previous studies have examined the role of CD36 in renal disease, metabolic disease, and others. For example, several studies have shown that CD36 participates in atherosclerotic arterial lesion formation by promoting oxidized LDL uptake in macrophages [32,33]. CD36 has also been implicated in insulin resistance [34], which is linked to diabetes. Moreover, a number of previous studies have examined whether CD36 is associated with fatty liver disease [35,36]. In this study, we found that CD36 was high expression in both glomerulus and renal tubular in the rats fed with high-fat diet. Moreover, in glomerulus, CD36 was co-located with nephrin, a podocyte-specific transmembrane protein, suggesting that CD36 was expressed in podocytes. In *in vitro* experiments, podocytes was stimulated with PA, a saturated LCFA, simulating the conditions of hyperlipidemia. The results showed that PA markedly upregulated CD36 expression (53, 78, and 88 kDa) in podocytes, trapping fatty acids within podocytes. This inappropriate CD36-mediated accumulation of excess lipids leads to cellular dysfunction and death, which is called lipotoxicity [37,38]. Lipotoxicity can affect actin cytoskeleton organization in podocytes through excess lipid accumulation [3,39]. Furthermore, PA mediated excess lipid accumulation and actin filament rearrangement throughout the podocyte cytoplasm, resulting in podocyte FP effacement. In contrast, SSO specifically inhibited CD36, suppressed PA-induced lipid accumulation, and restored actin cytoskeleton disruption, showing that CD36-mediated lipotoxicity participated in PA-induced actin cytoskeleton disruption.

Lipid overload contributes to mitochondrial dysfunction and oxidative stress [11,40]. ROS production was considerably increased in response to PA in this study. Oxidative stress is involved in the pathological processes of various diseases, including diabetes and atherosclerosis [41]. ROS generation could activate TRPC6, a downstream target of angiotensin II receptor signaling, in podocytes [42]. Palmitate-induced dysregulation of intracellular Ca^2+^ might link cytoskeleton rearrangements, and TRPC6 plays an important role in regulating Ca^2+^ homeostasis in podocytes [43]. Overexpression or gain of the TRPC6 channel is associated with actin cytoskeleton reorganization and drives podocyte FP effacement, inducing proteinuria [44]. Therefore, fatty acids may dynamically remodel the actin cytoskeleton of podocytes via the ROS-activated TRPC6 channel, resulting in changes in podocyte FP structure [45]. In this study, podocytes were treated with SSO, and the results showed that SSO significantly reduced PA-induced ROS production, further rescuing actin cytoskeleton disruption. Further experiments also showed that the potent antioxidant NAC ameliorated palmitate-induced rearrangement of the actin cytoskeleton, demonstrating that oxidative stress was involved in lipotoxicity-induced FP effacement mediated by CD36.

Lipid-lowering therapy has been shown to contribute to the reduction of the albumin excretion rate, subsequently improving renal function [46,47]. ANGPTL3, identified as a promising lipid-lowering therapy for atherosclerotic cardiovascular disease through the regulation of lipoprotein metabolism and lipid levels [48], may also serve as a potential therapeutic target for kidney injury mediated by dyslipidemia.

In conclusion, the scavenger receptor CD36, which is a common receptor for fatty acids, was shown to mediate excess lipid accumulation in podocytes, further promoting oxidative stress and ultimately resulting in podocyte FP effacement, which may occur through TRPC6 channel-activated actin reorganization. This is the first study to offer clinical and laboratory evidence of the participation of CD36 in lipotoxicity-induced podocyte FP effacement, suggesting that CD36 could be a therapeutic target for kidney damage accompanied by hyperlipidemia.

## 5 Conclusion

This study aimed to show that PA induced CD36 expression, lipid absorption, cytoskeletal rearrangement, and FP effacement. To gain further insight into the mechanisms by which CD36 mediates PA-induced alterations in podocyte morphology, ROS generation was suppressed with the potent antioxidant NAC to alleviate PA-induced podocyte FP effacement. This study showed that CD36-mediated oxidative stress was implicated in PA-induced FP effacement and may act as a novel therapeutic target for treating kidney damage accompanied by hyperlipidemia. However, this study had only conducted at the animal and cellular levels, and had not yet begun any clinical trials.
